# Anomalous Left Main Coronary Artery Arising from the Right Sinus of Valsalva in a Young Man Presenting with Recurrent Syncope and Myocardial Infarction

**DOI:** 10.1155/2018/9805061

**Published:** 2018-07-31

**Authors:** Koroush Khalighi, Munish Sharma, Amit Toor, Rubinder S. Toor, Gary Costacurta

**Affiliations:** ^1^EP Lab, Easton Hospital, Drexel University, Easton, PA, USA; ^2^Department of Cardiology, Easton Cardiovascular Associates, Easton, PA, USA; ^3^Department of Internal Medicine, Easton Hospital, Easton, PA, USA; ^4^Department of Public Health, Milken Institute School of Public Health, George Washington University, Washington, DC, USA

## Abstract

A 19-year-old man with the left main coronary artery (LMCA) arising from the right sinus of Valsalva presented with recurrent episodes of syncope and myocardial infarction (MI). Anomalous aortic origin of a coronary artery (AAOCA) is an uncommon but extremely important differential diagnosis that should not be missed in patients presenting with syncope, MI, ventricular arrhythmias, or cardiac arrest. A definitive diagnosis with coronary angiography and prompt surgical intervention is imperative in such symptomatic patients.

## 1. Introduction

Left main coronary artery (LMCA) or left anterior descending coronary artery (LAD) arising from the right sinus of Valsalva or right coronary artery (RCA) is referred to as an anomalous aortic origin of a coronary artery (AAOCA). The subsequent course is mostly between the aorta and the pulmonary artery on its way to the left ventricle, which could compress the vessel and may give rise to fatal outcomes like myocardial ischemia, ventricular arrhythmias, and sudden cardiac death [1, 2]. This is an uncommon case of LMCA arising from the right sinus of Valsalva in a 19-year-old man with recurrent episodes of syncope due to myocardial ischemia and infarction.

## 2. Case Description

A 19-year-old African American man was brought to the emergency department (ED) by emergency medical services (EMS) after an episode of syncope earlier on the day of admission while playing basketball at his college. This occurred suddenly and was associated with a transient episode of lightheadedness, diaphoresis, and blurred vision. This was followed by lost consciousness for less than 10 seconds, with spontaneous recovery as witnessed by his friends on the sidelines. There was not any involuntary movement of the body, urinary or bowel incontinence, or postictal confusion as per the witnesses. After regaining consciousness, there was a complaint of nonradiating, substernal, burning chest pain with a “6 out of 10” intensity, which lasted approximately 20–30 minutes and was relieved with a nitroglycerin sublingual pill given by EMS. On arrival to the ED, the patient was asymptomatic. Blood pressure was 103/67 mmHg, heart rate 85 bpm and regular, afebrile oxygen saturation > 95% on room air, and respiratory rate 12 per minute. Physical examination findings revealed a supple neck with no jugular venous distention; no carotid bruits were audible. Cardiovascular examination revealed a regular heart rhythm with normal S1 and S2 and no significant audible murmurs, parasternal heave, or thrill. The lungs were clear to auscultation bilaterally. There was no pitting pedal edema, and all peripheral pulses were palpable. The abdomen was soft and nondistended; no focal neurological deficits were evident.

History was positive for two similar events in the past. The first episode occurred approximately 10 years ago in Nigeria while playing soccer, and another event occurred a year ago year while running a block to catch a bus. Medical attention was not sought on both occasions. Family history was negative for similar syncopal attacks, sudden cardiac arrest, or arrhythmias. Social history was positive for drinking heavy amounts of Vodka with friends occasionally on the weekends along with marijuana but negative for cigarette smoking or other illicit drug use.

**Figure 1:**
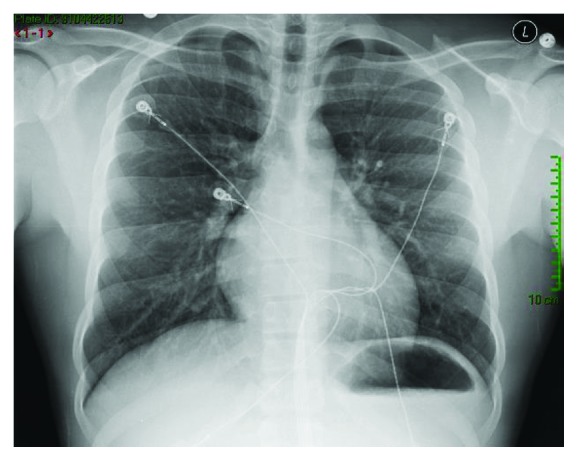
Chest X-ray did not reveal any significant radiographic abnormalities.

**Figure 2:**
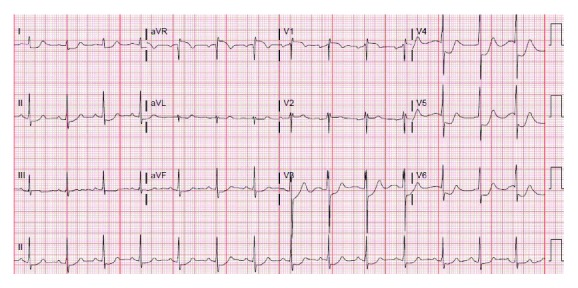
Admission EKG showed a rhythm of sinus origin at a rate of 80 BPM, with normal axis and 2-3 mm horizontal dawn sloping with ST segment depression in anterolateral leads.

**Figure 3:**
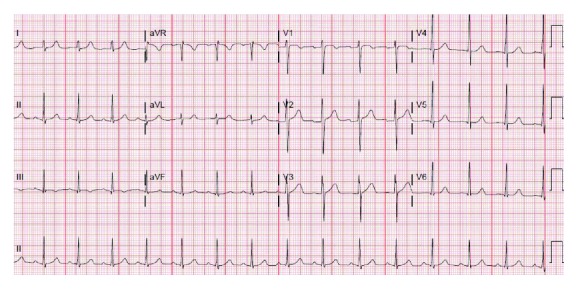
EKG 2 hours after admission showed a rhythm of sinus origin at a rate of 95 BPM, with normal axis and with reversal of ST-T wave changes present on admission.

**Figure 4:**
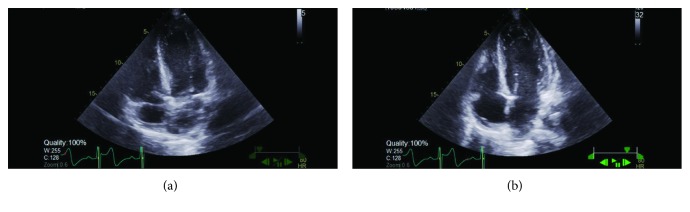
Two-dimensional transthoracic echocardiogram showing normal sized cardiac chambers. Diastole phase (b) and systole phase (a).

Initial laboratory values in the ED showed hemoglobin of 12.3 gm %, serum creatinine of 1.4 mg/dl (normal reference range 0.50 to 1.20 mg/dl) and BUN of 24 mg/dl, elevated initial troponin I of 0.23 ng/ml (normal reference range 0.00 to 0.07 ng/ml), total creatinine kinase level of 585 units/liter (normal reference range: 49 to 397 U/l), and urine with 11–20 hyaline casts. Thyroid stimulating hormone, serum potassium, and serum magnesium were within normal limits. His chest X-ray did not reveal any acute cardiopulmonary process (Figure 1). His initial electrocardiogram (EKG) revealed significant 4 mm to 5 mm ST depression in the anterolateral leads but otherwise unremarkable (Figure 2). A repeat EKG done within 2 hours of the initial presentation showed reversal of ST depression (Figure 3). A repeat troponin done in 6 hours revealed staggering troponin I of 53.30 ng/ml. Immediate bedside echocardiogram showed a nondilated left ventricular cavity with normal LV wall thickness and a mildly diminished LV ejection fraction at 45–50% (Figure 4). In the emergency room, immediate treatment with 325 mg of Aspirin and therapeutic Lovenox (1 mg/kg) was given. Overnight, periodic 5–7-beat runs of nonsustained ventricular tachycardia on the cardiac monitor occurred. Cardiac tomography (CT) angiography of the heart revealed a common trunk for the right and left coronary arteries arising from the right coronary cusp. The right coronary artery had a normal course. The LMCA traveled posteriorly between the ascending aorta and pulmonary outflow tract before resuming its normal course.

**Figure 5:**
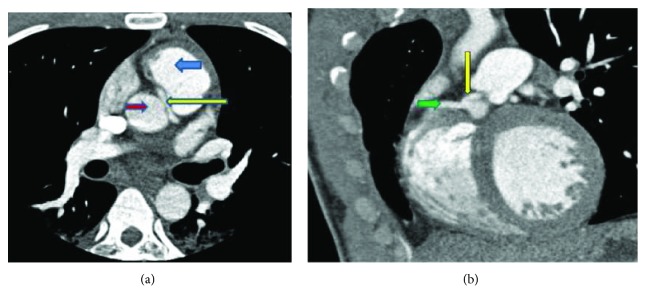
(a) Anomalous LMCA course (yellow arrow) between the aorta (red arrow) and the pulmonary outflow track (blue arrow) showing narrowing. (b) Oblique view demonstrating the common origin of the right (green arrow) and the left coronary arteries (yellow arrow).

**Figure 6:**
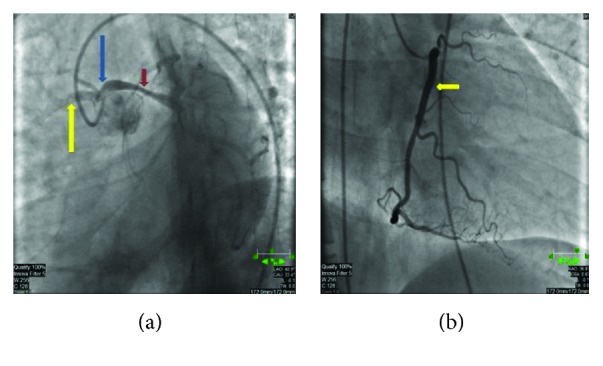
(a) demonstrates the anomalous origin of the left main coronary artery (blue arrow) along with stenosis of the left main coronary (red arrow). (b) demonstrates the course of the RCA (yellow arrows).

The LMCA narrowed in its midportion as it passed between these two structures (Figures 5(a) and 5(b)). These findings were confirmed by cardiac catheterization which showed an anomalous origin of the LMCA, originating from the right sinus of Valsalva (Figures 6(a) and 6(b)). The LMCA narrowed in its midportion with over 60–70% luminal stenosis due to a stricture present between the ascending aorta and the left ventricular outflow track. Afterwards, medical stabilization was achieved and a transfer to a tertiary care center was carried out.

Upon arrival at the tertiary care center, a procedure was performed involving the unroofing of the anomalous aortic origin of the left main coronary artery, including retro pulmonary unroofing by a congenital cardiothoracic surgeon. Recovery was satisfactory after the surgery, and postoperative complications did not occur.

## 3. Discussion

The overall incidence of the abnormal aortic origin of the coronary arteries is estimated to be approximately 0.64% of births [3]. The most common anomaly is the origin of the left circumflex artery from the right sinus of Valsalva, followed by the origin of a single coronary artery from the left sinus of Valsalva, origin of both the right and the left coronary arteries from the right sinus of Valsalva, and the LAD originating from the right sinus of Valsalva. The prevalence of the RCA arising from the left sinus of Valsalva is approximately 0.17% while the rarest anomaly is the left coronary artery arising from the right sinus of Valsalva and its prevalence is around 0.047% [4]. There are four major variations in the course taken by the LMCA after its origin from the right sinus of Valsalva, in relation to the aorta and the pulmonary trunk, on its way to the left ventricle. The LMCA can have a septal course beginning at the right ventricular infundibulum; it can have an anterior course, a retroaortic course, or an interarterial course between the aorta and the main pulmonary artery [5, 6].

The clinical presentation in AAOCA is commonly anginal chest pain or syncope that occurs mostly with exercise or other strenuous activities. In young athletes and military recruits, the first clinical presentation may unfortunately be sudden cardiac death [7]. In a registry of sudden cardiac death in 286 athletes, the anomalous coronary artery arising from the opposite sinus of Valsalva was the second most common cause of death (13%) besides hypertrophic cardiomyopathy [8]. Though the incidence of sudden cardiac death in patients with AAOCA is low overall, it is still comparatively higher for patients with AAOCA of the left coronary artery than with AAOCA of the RCA, especially in the absence of symptoms [9].

The pathophysiology behind syncope, myocardial ischemia, and sudden cardiac death has been postulated to be the compression of the anomalous coronary artery between the aorta and the pulmonary artery on its way to the left ventricle [1, 2, 10]. The intramural course and acute angle of take-off may predispose the affected artery to the obstruction of blood flow [11]. It has been proposed that there is an expansion of the aortic root and pulmonary trunk during exercise or strenuous physical activities which worsens the preexisting angulation of the coronary artery and thus reduces the diameter of the lumen in the proximal portion of the coronary artery [1]. The 19-year-old man might have had recurrent episodes of syncope and myocardial infarction on admission due to the compression of the interarterial portion of the LMCA between the aorta and the main pulmonary artery during strenuous physical activities.

Diagnosis is mainly based on the high index of clinical suspicion for the anomalous origin of the coronary artery. Coronary magnetic resonance angiography (CMRA) and coronary computed tomographic angiography (CCTA) are important noninvasive diagnostic tools that are being increasingly used to detect such cases. Several smaller studies have proven the efficacy of CMRA and CCTA [12, 13]. Coronary angiography is the gold standard for the diagnosis and evaluation of the origin and the course of anomalous coronary arteries and is particularly important when the noninvasive test fails to yield definitive results [14]. In patients with symptoms arising from ventricular tachyarrhythmia or myocardial infarction, surgical intervention is indicated, particularly in cases of the origin of the left coronary artery from the right sinus of Valsalva. The surgical intervention can consist of coronary artery bypass grafting (CABG) or unroofing (marsupialization) of the coronary artery that prevents further compression of the artery in its interarterial course [15]. For patients without any intramural course, the “hinge twist” procedure has been described [16]. Pulmonary artery translocation and intracoronary stents to prevent vessel compression are a few other alternative surgical procedures that have been described so far [17]. Benefits of surgical treatment in asymptomatic individuals with AAOCA have not been well established.

## 4. Conclusion

The left coronary artery arising from the right sinus of Valsalva is not a frequently encountered congenital coronary artery anomaly. In younger patients presenting with symptoms of syncope, myocardial ischemia, or ventricular arrhythmias, a high index of clinical suspicion is required. This man received a diagnosis at the age of 19 years despite having prior symptoms. A future fatal outcome may have occurred if anomalous coronary artery disease was not detected and treated promptly. Definitive diagnosis with coronary angiography and prompt surgical intervention is imperative in such symptomatic patients. Data regarding outcomes of surgical intervention in asymptomatic patients are still lacking. Noninvasive tests such as echocardiography and computed tomography angiography can aid in diagnosis and are becoming increasingly popular.
